# Supplementary material for: Genetic diversity varies with species traits and latitude in predatory soil arthropods (Myriapoda: Chilopoda)
Source: Glob Ecol Biogeogr. Author manuscript; Available in PMC 2024 May 3. (PMC7615927; doi:10.1111/geb.13709)
Supplement: Appendix S3 [file EMS195648-supplement-Appendix_S3.docx]

**Table S3.1.** Summary of sequence statistics, taxonomic information, species traits and biogeographic variables in the compiled dataset presented as a mean value across species. For columns summarising numerical data, the mean is provided along with the range of observed values within parentheses. The total number of valid species in each order (from Edgecombe & Giribet, 2007) is provided in parantheses against the number of species present in the current analysis.

|  | **Overall** | **Craterostigmomorpha** | **Geophilomorpha** | **Lithobiomorpha** | **Scolopendromorpha** | **Scutigeromorpha** |
| --- | --- | --- | --- | --- | --- | --- |
| **No. families** | **13** | 1 | 3 | 2 | 5 | 2 |
| **No. species** | **128** | 2  (2) | 17  (1300) | 40  (1100) | 64  (800) | 5  (100) |
| **Mean number of sequences per species** | **10**  **(3 - 68)** | 42  (17 - 68) | 6  (3 - 19) | 8  (3 - 26) | 11  (3 - 66) | 3  (3 - 5) |
| **Mean alignment length per species (bp)** | **649**  **(465 - 840)** | 750  (745 - 756) | 613  (495 - 658) | 651  (609 - 676) | 646  (465 - 840) | 743 (656 - 811) |
| **Mean number of unique locations per species** | **8**  **(1 - 53)** | 12  (10 - 14) | 5  (2 - 18) | 7  (1 - 20) | 9  (1 - 53) | 3  (2 - 4) |
| **Average pairwise difference per species** | **0.07**  **(0.00 - 0.17)** | 0.11  (0.11 - 0.12) | 0.06  (0.00 - 0.15) | 0.06  (0.00 - 0.16) | 0.08  (0.00 - 0.17) | 0.08  (0.02 - 0.13) |
| **Mean body size per species (mm)** | **48.05**  **(8.50 - 250.00)** | 43.50  (37.00 - 50.00) | 44.12  (11.00 - 95.00) | 17.50  (8.50 - 48.00) | 70.41  (20.00 - 250.00) | 21.40  (15.00 - 27.00) |
| **Maternal care: Present** | **83 / 128** | 2 / 2 | 17 / 17 | 0 / 40 | 64 / 64 | 0 / 5 |
| **Vision: Present** | **98 / 128** | 2 / 2 | 0 / 17 | 40 / 40 | 51 / 64 | 5 / 5 |
| **Mean latitudinal range per species (degrees)** | **15.95**  **(0.00 - 71.66)** | 7.38  (2.31 - 12.45) | 15.77  (3.40 - 28.16) | 17.93  (0.00 - 37.41) | 15.76  (0.00 - 71.66) | 6.49  (0.65 - 15.81) |
| **Mean latitude of sequence data per species (degrees)** | **24.72**  **(-42.41 - 57.45)** | -41.93  (-42.41 - -41.44) | 44.99  (22.98 - 57.45) | 42.13  (-41.50 - 51.95) | 14.71  (-34.66 - 50.25) | -28.55  (-33.98 - -21.26) |
| **Mean geographic distance between sequences per species (km)** | **439.96**  **(0.00 - 5,065.76)** | 250.54  (116.74 - 384.33) | 336.38  (0.10 - 1,490.11) | 304.40  (0.00 - 1,988.20) | 575.47  (0.00 - 5,065.76) | 217.71  (26.61 - 580.79) |

Edgecombe, G. D., & Giribet, G. (2007). Evolutionary biology of centipedes (Myriapoda: Chilopoda). Annual Review of Entomology, 52, 151–170. https://doi.org/10.1146/annurev.ento.52.110405.091326


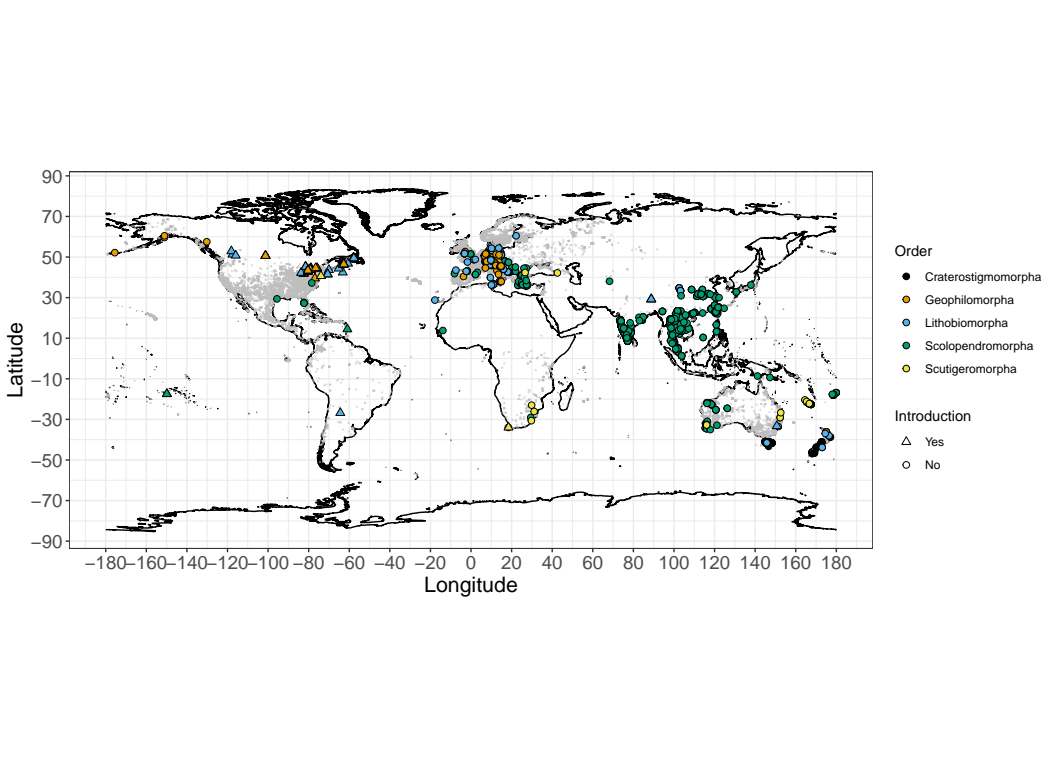


**Figure S3.1.** Geographic locations of centipede species associated with at least three mitochondrial COI sequences. Filled circles are data used to calculate genetic diversity and used in further analysis, while triangles represent data associated with likely introductions that were not considered in the main analysis. The grey circles are GBIF occurrences for centipedes across the globe.
