# Supplementary material for: Genetic diversity varies with species traits and latitude in predatory soil arthropods (Myriapoda: Chilopoda)
Source: Glob Ecol Biogeogr. Author manuscript; Available in PMC 2024 May 3. (PMC7615927; doi:10.1111/geb.13709)
Supplement: Appendix S5 [file EMS195648-supplement-Appendix_S5.docx]

**Appendix S5.**  **Additional beta regression models**

**A. Analysis including synanthropic introductions**

The complete dataset, including records representing likely synanthropic introductions, consisted of six additional species (total of 134 species) with changes to the number of sequences and/or species latitudinal range for seven species common to both the datasets. In this dataset, the largest change in predictor variables was seen in average geographic distance between sequences, ranging up to 9586 km, and species latitudinal range, ranging up to 103.22 degrees. In terms of longitudinal distribution of data, likely introductions of European species into the Americas were retained in this dataset, and these areas had very few sequence representatives. With respect to species traits, there were 85 species showing maternal care and 101 species with vision. In total, this dataset represented sequences from 834 unique locations and the mean genetic diversity across species was comparable to the analysis with introduced species excluded, at a value of 0.0703 (range = 0 to 0.1713).

In the analysis carried out using this dataset, the model using fixed effects with a precision parameter had the lowest AIC score, explaining 14.64% variation in genetic diversity across species (Table S5.2). However, while the nature of relationship of life history traits (body size and maternal care) and mean latitude with genetic diversity remained the same, vision emerged as a significant morphological trait, showing a positive relationship with genetic diversity. The average geographic distance between sequences was not a significant predictor (Figure S5.2).

**Table S5.2.** Parameter estimates and bootstrapped confidence intervals from the best performing beta regression model -

Genetic diversity_i_ ~ Beta(*μ_i_, ϕ_i_*)

logit(*μ_i_*) = Body size*_i_* + Vision*_i_* + Maternal care*_i_* + Mean latitude*_i_* + Latitudinal range*_i_ +* Average geographic distance*_i_*

*ϕ_i_ ~* Number of sequences*_i_*

The input dataset retained sequences and range extent from likely synanthropic introductions for species.

| **Parameters** | **Estimate*^1^*** | **SE*^2^*** | **Bootstrap 95% CI** | **z-value** |
| --- | --- | --- | --- | --- |
| **Mean** | | | | |
| **Intercept** | -3.382*** | 0.239 | -3.854 - -2.784 | -14.135 |
| **Mean latitude** | -0.196** | 0.062 | -0.319 - -0.071 | -3.150 |
| **Average geographic distance** | 0.072 | 0.086 | -0.156 - 0.378 | 0.843 |
| **Species latitudinal range** | 0.031 | 0.087 | -0.194 - 0.280 | 0.351 |
| **Body size** | -0.260** | 0.080 | -0.512 - -0.052 | -3.266 |
| **Vision: Yes** | 0.513** | 0.174 | 0.052 - 0.897 | 2.942 |
| **Maternal care: Yes** | 0.653*** | 0.186 | 0.244 - 1.049 | 3.506 |
| **Precision** | | | | |
| **Intercept** | 3.164*** | 0.127 | 3.045 - 3.504 | 25.002 |
| **Number of sequences** | 0.279* | 0.117 | 0.094 - 1.273 | 2.373 |
| Pseudo R-squared = 0.1464  Log-likelihood = 239.0542  N = 134  *^1^* *** = p < 0.001, ** = p < 0.01, * = p < 0.05, . = p < 0.1 *^2^* Standard error | | | | |


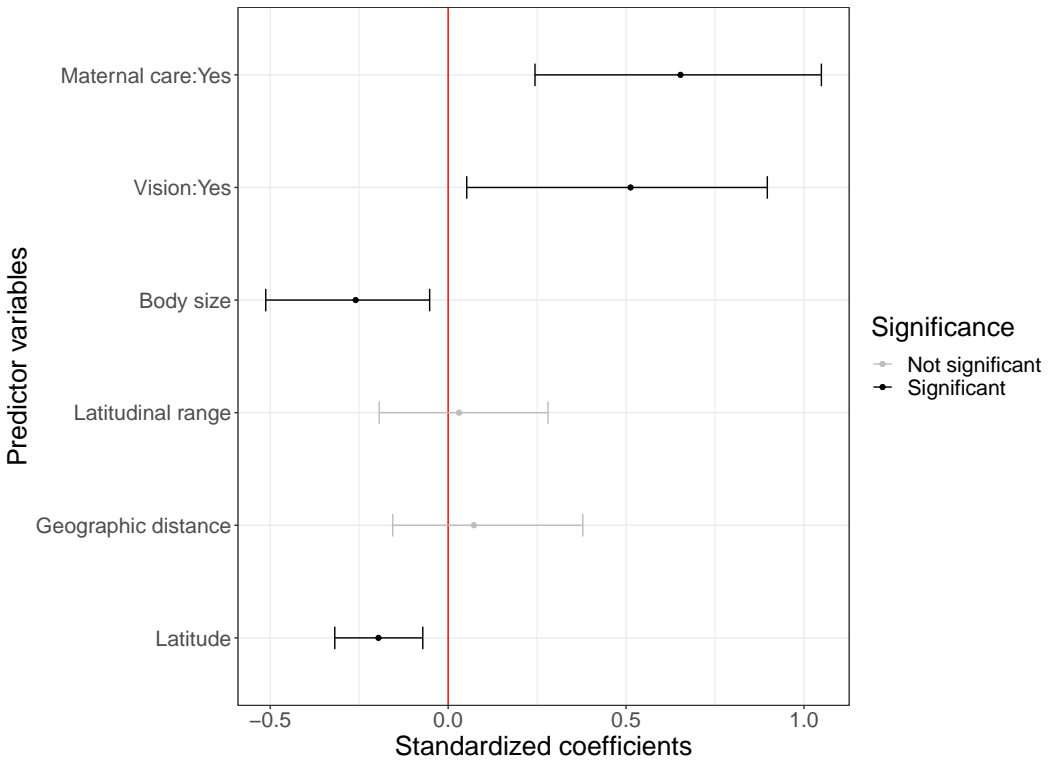


**Figure S5.2.** Standardized coefficient estimates (logit-scale) from the beta regression model with the lowest AIC value specified as -

*Genetic Diversity ~ Body size + Vision + Maternal care + Mean latitude + Latitudinal range* + *Geographic distance | Number of sequences*

The input dataset retains sequences and range extent corresponding to potential synanthropic introductions. Mean coefficient estimates are represented as points and their bootstrapped 95% confidence intervals are displayed as error bars for each predictor variable. Positive values indicate a positive relationship between the corresponding predictor variable and genetic diversity and the converse.

**B. Analysis accounting for spatial autocorrelation in model residuals**

**Background**

Spatial autocorrelation can arise due to endogenous processes (Gaspard et al., 2019), such as dispersal limitation, which leads to greater similarity of biological phenomena at sites that are closer to each other. For example, individuals of a species sampled from nearby locations are expected to show greater sequence similarity as a result of relatively frequent dispersal and gene flow as compared to distantly located individuals. Such sources of spatial autocorrelation are grouped under distance-related processes (Dormann et al., 2007).

Alternately, spatial autocorrelation in biological phenomena can arise if the exogenous mechanisms generating them are spatially correlated. For example, if climatic and historic factors shaping genetic diversity show greater similarity at shorter distances, this spatial autocorrelation would be carried over to the resulting patterns in genetic diversity. The spatial autocorrelation in a response variable resulting from a spatially structured predictor is known as induced spatial dependence (Dormann et al., 2007). Apart from these processes, aspects of sampling design such as grain size and scale, or methodological choices related to the specification of the shape of relationship between the predictor and response can lead to residual spatial autocorrelation (Dormann et al., 2007; Gaspard et al., 2019).

Spatial autocorrelation in model residuals can lead to violation of the assumption of regression analysis, where observations are considered independent of each other. Therefore, the number of data points used in the analysis would not be a true indicator of sample size, leading to inflated degrees of freedom. This can result in high false positive rates, leading to the detection of spurious relationships, when they are not supported by the data at hand. Therefore, the presence of significant spatial autocorrelation in model residuals can have important consequences on model results and the inferences made using them (Dormann et al., 2007; Gaspard et al., 2019).

Spatial autocorrelation in model residuals can be thought of in two different ways – as an opportunity to understand the processes generating the pattern, which can be biologically meaningful, or as a challenge that impedes the process of hypothesis testing leading to unreliable results (Dormann et al., 2007). There are several methods of dealing with spatial autocorrelation in model residuals, which fall into two broad categories – those that use additional covariates to capture it and spatially explicit models that consider spatial processes within the modeling framework (Dormann et al., 2007).

**Methodological approach**

In the beta regression model, we incorporated predictors to account for endogenous and exogenous processes that may lead to spatial autocorrelation. We used average distance between sampled sequences as a predictor of the genetic diversity estimate for a species, where greater geographic distance between individuals would lead to higher values of genetic diversity as a result of isolation due to limited dispersal. We used latitude as a proxy for climatic and historic processes shaping genetic diversity. We expect lower latitudes to have greater climatic stability over time leading to more stable populations and higher genetic diversity. Higher temperature at lower latitudes can also result in higher genetic diversity due to higher metabolic and mutation rates. Therefore, species found at similar latitudes with overlapping ranges are expected to show a significant correlation in genetic diversity, all things being equal.

To check if the predictors described above were sufficient in accounting for the spatial autocorrelation in genetic diversity, we tested for the presence of spatial autocorrelation in model residuals. We calculated the centroid of sequence coordinates (mean latitude and longitude) for each species, and created a matrix of haversine distances between all pairs of species centroids. We used the inverse of this matrix to calculate Moran’s *I* using the response model residuals in a two sided hypothesis test. We found small but significant (Moran’s *I* = 0.0726, *p* = 0.0083) spatial autocorrelation in model residuals, calculated using the function *Moran.I* in the package ‘ape’ (Paradis & Schliep, 2019). Correlograms of local Moran’s *I* using the distance-based (function *correlog* in package ‘ncf’ – Bjornstad, 2022; 500 km intervals) method showed significant positive and negative values at various distance classes.

To retain the beta regression analysis framework, we chose to use spatial eigenvectors as additional predictors in our model. Spatial eigenvectors are obtained from the diagonalization of a doubly centred spatial weighing matrix, and may represent spatial patterns at multiple scales (Bauman et al., 2018a). Spatial eigenvectors with large positive values represent high positive spatial autocorrelation and global spatial structures, while large negative values represent high negative autocorrelation and local spatial structures (Dray et al., 2006). The incorporation of spatial eigenvectors is flexible to the model’s error distribution and applicable even when there is non-stationarity and anisotropy in the pattern of spatial autocorrelation (Dormann et al., 2007). Using this approach, we removed the spatial autocorrelation in the model residuals and evaluated if there was a significant change in coefficient estimates and our inferences. We optimized the selection of a spatial weighting matrix following the recommendations of Bauman et al. (2018a) and good practices in model selection suggested by Bauman et al. (2018b).

As graph-based methods of creating a connectivity matrix have been shown to consistently perform better that distance-based methods across different sampling schemes and types of spatial autocorrelation, we compared spatial weighting matrices derived from three best performing graph based methods as per Bauman et al. (2018a) – Gabriel, Relative neighbourhood and Minimum Spanning Tree (Figure S5.3). The input data for these graphs were species centroids described above. Each of these three neighbourhood graphs were either left unweighted (binary) or weighted with a linear distance-based weighting function to generate six different spatial weighting matrices. To optimize the selection of spatial eigenvectors by comparing these matrices, we used the criterion of minimization of Moran’s *I* in the model residuals. This procedure selects the minimum number of spatial eigenvectors that render the spatial autocorrelation in model residuals not significant (Bauman et al. 2018b). This optimization procedure revealed that two spatial eigenvectors from a binary spatial weighting matrix derived from the Relative Neighbourhood graph were the best choice of spatial predictors (Figure S5.4). The selection of spatial eigenvectors was optimised using functions from ‘spdep’ package (Bivand & Wong, 2018). On incorporating the two spatial eigenvectors into our beta regression model, residual spatial autocorrelation was found to be not significant (Moran’s *I* = 0.069 *p* = 0.0111) when considering a critical value of 0.01 (default value in the function) (Figure S5.5). The relationships between our original predictors and genetic diversity largely remained the same in this updated model, with wider confidence intervals for coefficient estimates of body size and maternal care from 1000 bootstrapped replicates (Figure S5.6, Table S5.3).


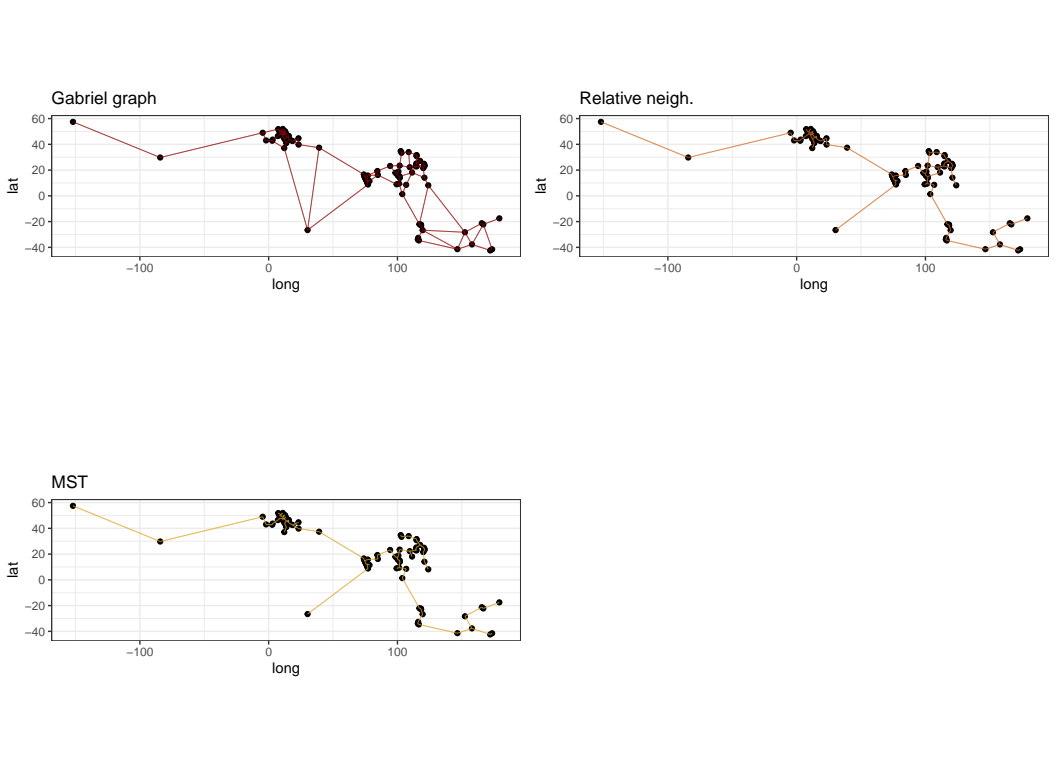


**Figure S5.3.** The three types of connectivity graphs used to create spatial weighting matrices. The spatial eigenvectors derived from these matrices were compared to select the subset that significantly reduces spatial autocorrelation in model residuals. Each point on a graph represents the centroid of sequence coordinates for a given species, and an edge between two points represents a connection between them. MST – minimum spanning tree


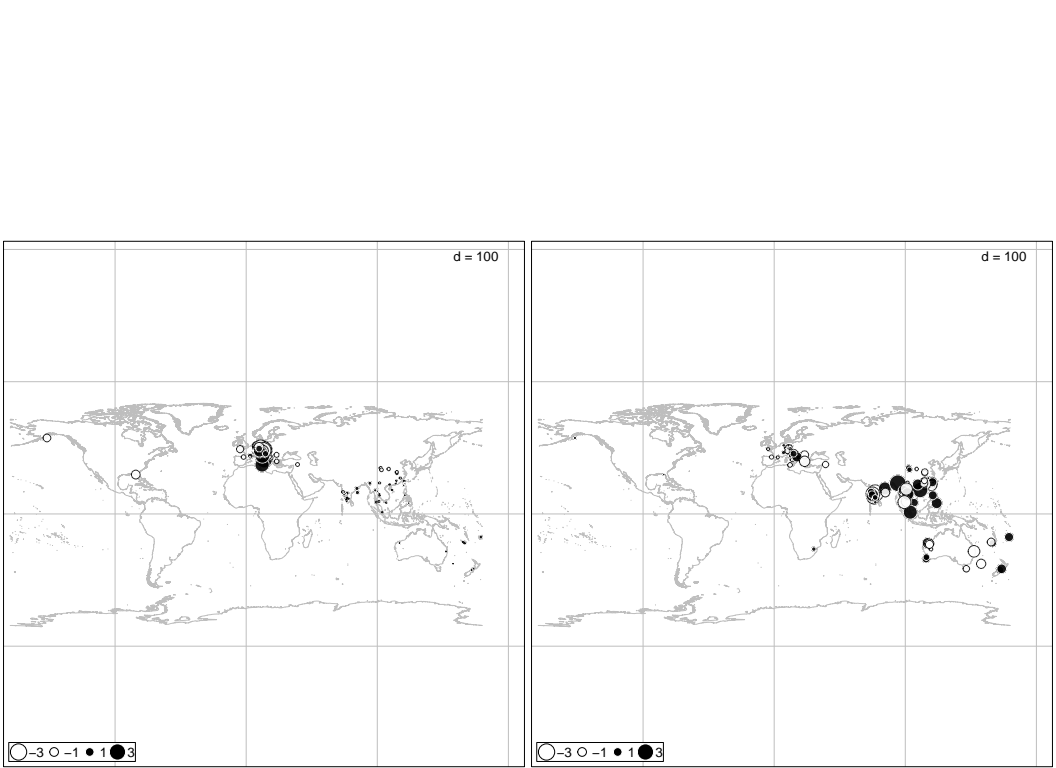
**Figure S5.4.** Plots of the two spatial eigenvectors, which were selected to account for residual spatial autocorrelation in the beta regression model. These spatial eigenvectors were derived from a spatial weighting matrix corresponding to a binary Relative Neighbourhood graph built using sequence centroids of each centipede species.


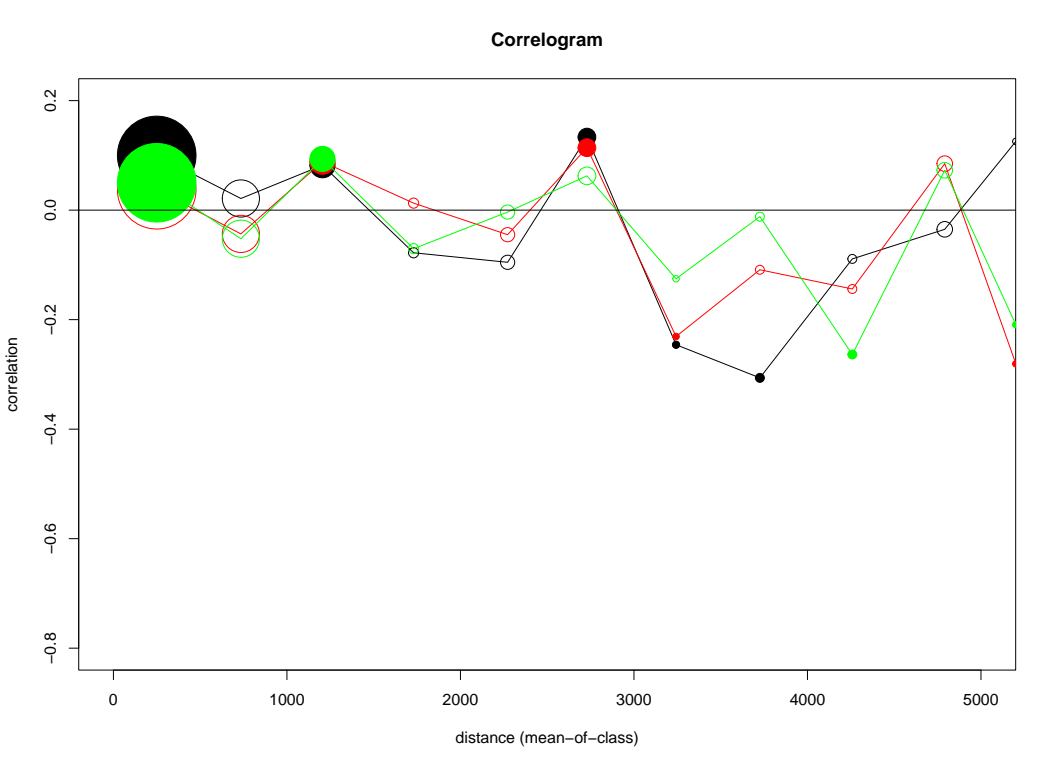


**Figure S5.5.** Correlogram of local Moran’s *I* at different distance classes (black – raw genetic diversity values, red – residuals from beta regression with only traits and biogeographic variables as predictors, green – residuals from beta regression with spatial eigenvectors along with traits and biogeographic variables as predictors). Filled circles represent significant values of Moran’s *I.* The size of a circle represents the number of data points falling into a particular distance class.


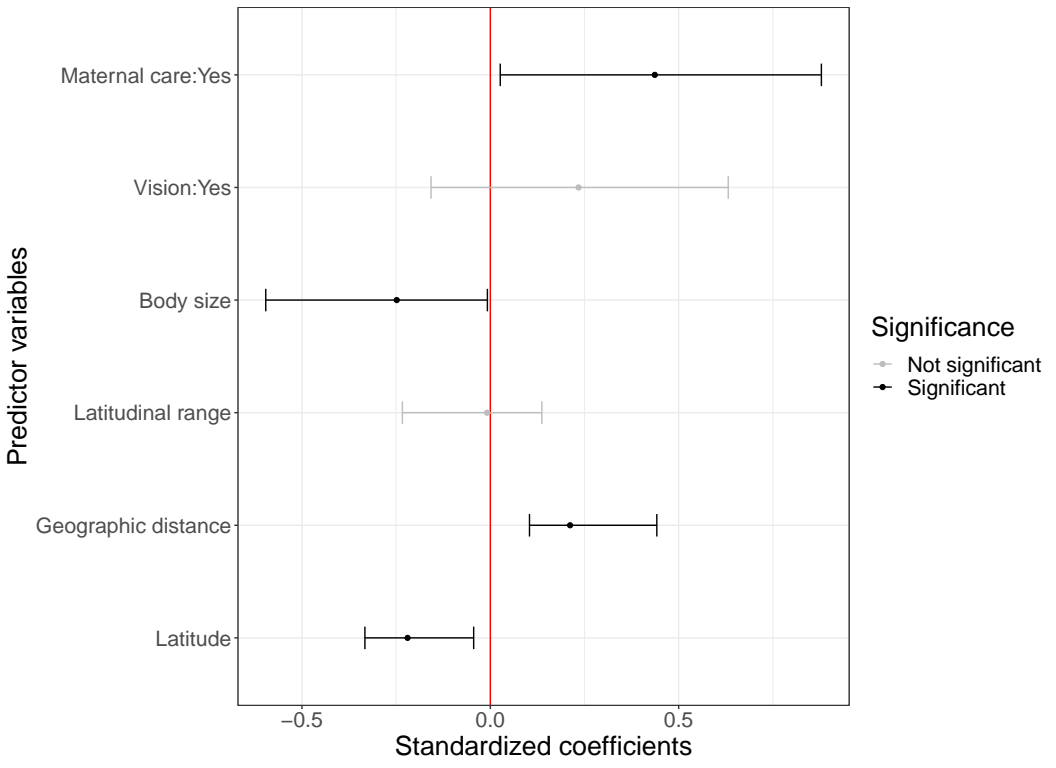


**Figure S5.6.** Standardized coefficient estimates (logit-scale) from the beta regression model with the lowest AIC value specified as -

*Genetic Diversity ~ Body size + Vision + Maternal care + Mean latitude + Latitudinal range + Geographic distance + MEM13 + MEM40 | Number of sequences*

The spatial eigenvectors (‘MEM’ in the predictors) were obtained from a spatial weighting matrix, which was a product of a connectivity matrix derived from a Relative Neighbourhood graph of coordinates (centroid of sequence locations for a species), and a binary weighting matrix. Mean coefficient estimates are represented as points and 95% confidence intervals obtained from 1000 bootstrapped replicates are displayed as error bars for each predictor variable. Positive values indicate a positive relationship between the corresponding predictor variable and genetic diversity and the converse.

**Table S5.3.** Parameter estimates (standardized and in the logit scale) from the best performing beta regression model using additional spatial eigen vectors as predictors to account for small residual spatial autocorrelation defined as -

Genetic diversityi ~ Beta(*μ_i_, ϕ_i_*)

logit(*μ_i_*) = Body size*_i_* + Vision*_i_* + Maternal care*_i_* + Mean latitude*_i_* + Latitudinal range*_i_ +* Average geographic distance*_i_ +* MEM13*_i_* + MEM40*_i_*

*ϕ_i_ ~* Number of sequences*_i_*

| **Parameters** | **Estimate*^1^*** | **SE*^2^*** | **Bootstrap 95% CI** | **z-value** |
| --- | --- | --- | --- | --- |
| **Mean** | | | | |
| Intercept | -3.061*** | 0.227 | -3.607 - -2.447 | -13.500 |
| Mean latitude | -0.220*** | 0.059 | -0.333 - -0.044 | -3.746 |
| Average geographic distance | 0.212*** | 0.061 | 0.104 - 0.442 | 3.462 |
| Species latitudinal range | -0.009 | 0.070 | -0.233 - 0.137 | -0.128 |
| Body size | -0.248** | 0.077 | -0.596 - -0.008 | -3.245 |
| Vision: Yes | 0.234 | 0.164 | -0.157 - 0.632 | 1.427 |
| Maternal care: Yes | 0.437* | 0.176 | 0.026 - 0.879 | 2.482 |
| MEM 13 | 0.199** | 0.061 | 0.034 - 0.365 | 3.268 |
| MEM 40 | -0.185*** | 0.053 | -0.269 - -0.061 | -3.468 |
| **Precision** | | | | |
| Intercept | 3.417*** | 0.129 | 3.296 - 3.870 | 26.523 |
| Number of sequences | 0.228. | 0.121 | 0.026 - 2.084 | 1.883 |
| Pseudo R-squared = 0.2757  Log-likelihood = 240.2305  N = 128  *^1^* *** = p < 0.001, ** = p < 0.01, * = p < 0.05, . = p < 0.1 *^2^* Standard error | | | | |

**C. Analysis informed by sensitivity analysis of sample size**

The estimate of genetic diversity for a species would be influenced by the number of individuals that have been sampled to obtain sequences. When repeated samples of individuals are drawn from a population and genetic diversity is calculated for each draw, the variance of the estimate across draws is expected to be higher for smaller sample sizes. If the sample of individuals drawn at random is a poor representative of the population, as in the case where sampling is not done systematically, it can lead to an biased estimate. Since we use publicly available sequence data for analysis where there is a wide variation in sample size across species, we used the number of sequences as a predictor of the precision of our genetic diversity estimate in the statistical model. We find that the number of sequences per species is a significant predictor of precision from the results of this model.

To further check if our results are sensitive to sample size, we performed sensitivity analysis following Barrow et al. (2021). For each species, we randomly sampled 2 to 10 sequences from the total set of available sequences. This was repeated 100 times for a given sample size, where genetic diversity was calculated in each iteration. The variance in genetic diversity was calculated across the 100 iterations for a given sample size and species. We obtained plots of variance in genetic diversity against sample size for all the species used in our analysis. Looking through these plots (Figure S5.7), we observed that the variance in genetic diversity decreased substantially at a sample size of 4-5 sequences. Based on these results, we used a cut-off of four sequences to select species for the beta regression analysis (n = 91 species), and used the median value of genetic diversity from the 100 iterations as the response variable. The median genetic diversity represented the estimate from the full dataset quite well, with a Pearson correlation coefficient of 0.96 (*p* < 0.001). Even with only 71% of the species retained in the analysis after applying the sequence cut-off, the broad results overlapped with those from the full dataset (Figure S5.8, Table S5.4). The differences from our main model results were that maternal care and average geographic distance had wider bootstrapped confidence intervals that overlapped with zero, where maternal care had a p-value of 0.07 and was not significant in the regression model.


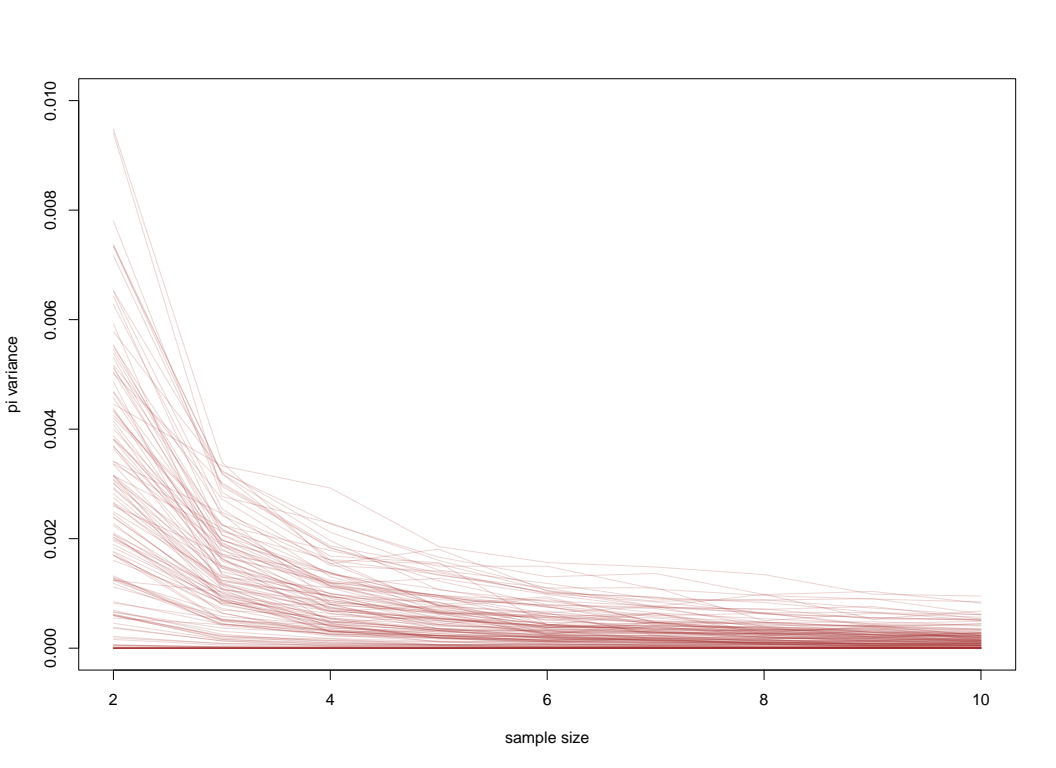


**Figure S5.7.** Scaling of variance in genetic diversity estimate across 100 replicates with sample size


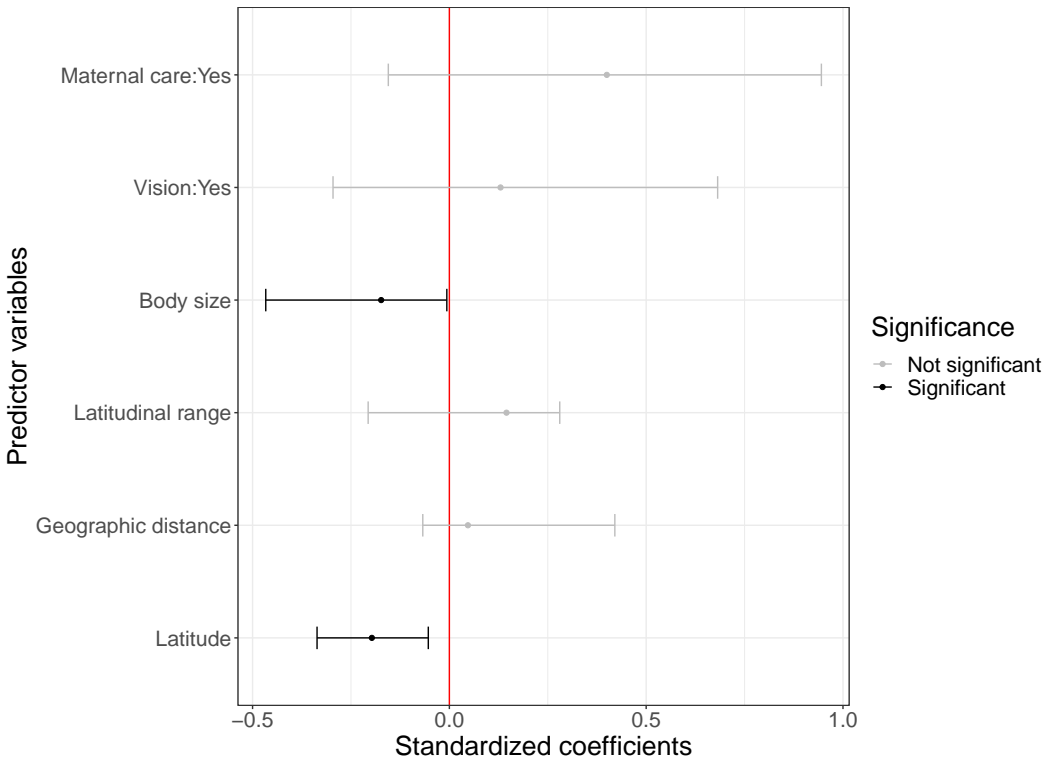


**Figure S5.8.** Standardized coefficient estimates (logit-scale) from the beta regression model with the lowest AIC value specified as -

*Genetic Diversity ~ Body size + Vision + Maternal care + Mean latitude + Latitudinal range + Geographic distance | Number of sequences*

Genetic diversity for a species is the median value obtained from 100 replicate draws, where four sequences were randomly sampled with replacement in each draw to calculate genetic diversity. This exercise was carried out for 91 species, which had at least four sequence representatives.

**Table S5.4.** Parameter estimates (standardized and in the logit scale) from the best performing beta regression using a more stringent sample size cut-off to select species, defined as -

Genetic diversity_i_ ~ Beta(*μ_i_, ϕ_i_*)

logit(*μ_i_*) = Body size*_i_* + Vision*_i_* + Maternal care*_i_* + Mean latitude*_i_* + Latitudinal range*_i_ +* Average geographic distance*_i_*

*ϕ_i_ ~ Number of sequences_i_*

Genetic diversity for a species is the median value obtained from 100 replicate draws, where four sequences were randomly sampled with replacement in each draw to calculate genetic diversity. This exercise was carried out for 91 species, which had at least four sequence representatives.

| **Parameters** | **Estimate*^1^*** | **SE*^2^*** | **Bootstrap 95% CI** | **z-value** |
| --- | --- | --- | --- | --- |
| **Mean** | | | | |
| **Intercept** | -2.887*** | 0.278 | -3.650 - -2.116 | -10.398 |
| **Mean latitude** | -0.197** | 0.061 | -0.336 - -0.054 | -3.239 |
| **Average geographic distance** | 0.047 | 0.076 | -0.068 - 0.420 | 0.621 |
| **Species latitudinal range** | 0.145. | 0.076 | -0.206 - 0.280 | 1.922 |
| **Body size** | -0.173* | 0.069 | -0.467 - -0.006 | -2.517 |
| **Vision: Yes** | 0.130 | 0.192 | -0.296 - 0.682 | 0.676 |
| **Maternal care: Yes** | 0.400. | 0.223 | -0.155 - 0.945 | 1.797 |
| **Precision** | | | | |
| **Intercept** | 3.522*** | 0.150 | 3.380 - 3.956 | 23.463 |
| **Number of sequences** | 0.541*** | 0.146 | 0.133 - 2.690 | 3.704 |
| Pseudo R-squared = 0.2141  Log-likelihood = 172.8361  N = 91  *^1^* *** = p < 0.001, ** = p < 0.01, * = p < 0.05, . = p < 0.1 *^2^* Standard error | | | | |

**References**

1. Barrow, L. N., Fonseca, E. M. da, Thompson, C. E. P., & Carstens, B. C. (2021). Predicting amphibian intraspecific diversity with machine learning: Challenges and prospects for integrating traits, geography, and genetic data. Molecular Ecology Resources, 21(8), 2818-2831. https://doi.org/10.1111/1755-0998.13303
2. Bauman, D., Drouet, T., Fortin, M.-J., & Dray, S. (2018a). Optimizing the choice of a spatial weighting matrix in eigenvector-based methods. Ecology, 99(10), 2159–2166. https://doi.org/10.1002/ecy.2469
3. Bauman, D., Drouet, T., Dray, S., & Vleminckx, J. (2018b). Disentangling good from bad practices in the selection of spatial or phylogenetic eigenvectors. Ecography, 41(10), 1638–1649. https://doi.org/10.1111/ecog.03380
4. Bivand, R. S., & Wong, D. W. S. (2018). Comparing implementations of global and local indicators of spatial association. TEST, 27(3), 716–748. https://doi.org/10.1007/s11749-018-0599-x
5. Bjornstad ON (2022). ncf: Spatial Covariance Functions. R package version 1.3-2, https://CRAN.R-project.org/package=ncf
6. Dormann, C., McPherson, J., Araújo, M., Bivand, R., Bolliger, J., Carl, G., Davies, R., Hirzel, A., Jetz, W., Kissling, W., Kühn, I., Ohlemüller, R., Peres-Neto, P., Reineking, B., Schröder, B., Schurr, F., & Wilson, R. (2007). Methods to Account for Spatial Autocorrelation in the Analysis of Species Distributional Data: A Review. Ecography, 30, 609–628. https://doi.org/10.1111/j.2007.0906-7590.05171.x
7. Dray, S., Legendre, P., & Peres-Neto, P. R. (2006). Spatial modelling: A comprehensive framework for principal coordinate analysis of neighbour matrices (PCNM). Ecological Modelling, 196(3), 483–493. https://doi.org/10.1016/j.ecolmodel.2006.02.015
8. Gaspard, G., Kim, D., & Chun, Y. (2019). Residual spatial autocorrelation in macroecological and biogeographical modeling: A review. Journal of Ecology and Environment, 43(1), 19. https://doi.org/10.1186/s41610-019-0118-3
9. Paradis, E., & Schliep, K. (2019). ape 5.0: An environment for modern phylogenetics and evolutionary analyses in R. Bioinformatics, 35(3), 526–528. https://doi.org/10.1093/bioinformatics/bty633
